# A High-Order, Symplectic, Finite-Difference Time-Domain Scheme for Bioelectromagnetic Applications within the Mother/Fetus Model

**DOI:** 10.1371/journal.pone.0114425

**Published:** 2014-12-10

**Authors:** YingJie Gao, HongWei Yang

**Affiliations:** Department of Physics, College of Science, Nanjing Agricultural University, Nanjing, Jiangsu, People’s Republic of China; Technion - Israel Institute of Technology, Israel

## Abstract

An explicit high-order, symplectic, finite-difference time-domain (SFDTD) scheme is applied to a bioelectromagnetic simulation using a simple model of a pregnant woman and her fetus. Compared to the traditional FDTD scheme, this scheme maintains the inherent nature of the Hamilton system and ensures energy conservation numerically and a high precision. The SFDTD scheme is used to predict the specific absorption rate **(**SAR) for a simple model of a pregnant female woman (month 9) using radio frequency (RF) fields from 1.5 T and 3 T MRI systems (operating at approximately 64 and 128 MHz, respectively). The results suggest that by using a plasma protective layer under the 1.5 T MRI system, the SAR values for the pregnant woman and her fetus are significantly reduced. Additionally, for a 90 degree plasma protective layer, the SAR values are approximately equal to the 120 degree layer and the 180 degree layer, and it is reduced relative to the 60 degree layer. This proves that using a 90 degree plasma protective layer is the most effective and economical angle to use.

## Introduction

Because the protection of a pregnant woman and her fetus is very important, the algorithm that is used in mother/fetus modeling and simulation must have a high precision and must be numerically stable.

The Finite Different Method (FDM), Finite Element Method (FEM) and Finite Volume Method (FVM) can be used for calculating the SAR values of the mother/fetus model. The FEM is more precise caused by the possibility to use high-order approximations. And since the methods for the high-order calculations of the FVM are developed by various groups recently [1], [2], the statement that high-order calculations are not available for the FVM does not hold true anymore at present time. Nevertheless, the FDM is better for processing the simplified geometries, the FEM and FVM are not necessary for the calculation of the mother/fetus model. Furthermore, the FDM can give simpler linear equations systems which could be solved faster. Also the finite-difference time-domain (FDTD) scheme is one of the FDM based on the time-domain. It can cover a wide frequency range with a single simulation and treat nonlinear material properties in a natural way. So the FDTD scheme is considered for our mother/fetus modeling and simulation.

To this end, our proposed high-order, symplectic, finite-difference time-domain (SFDTD) scheme is compared to the traditional FDTD scheme and the high-order FDTD scheme.

The traditional FDTD scheme has two shortcomings. First, it cannot accurately model curved, complex surfaces and material discontinuities by using the stair-casing approach with structured grids. Second, this scheme accumulates significant errors from numerical instability, dispersion and anisotropies.

To reduce the numerical dispersion in the traditional FDTD scheme, a variety of high-order spatial discretization strategies have been proposed [3], [4]. Based on orthonormal Harr wavelet expansions, a multi-resolution time-domain (MRTD) method [5], [6] was proposed. Another approach is the high-order FDTD scheme, which uses the Fang(2,4) [7] and Ty(2,4) [8] formats, retains the simplicity of the original Yee algorithm and can conserve computational resources by using coarse grids compared to the traditional FDTD scheme. In addition, the discrete singular convolution [9], [10] scheme was proposed, which uses delta cores, such as the Shannon core, Poisson core and the Lagrange core. However, these high-order approaches must use lower Courant-Friedrichs-Levy (CFL) numbers to comply with the stability criterion that subsequently destroys the symplectic structure of the electromagnetic system.

Because Maxwell’s equations can be written as an infinite-dimension Hamiltonian system, a stable and accurate solution can be obtained by using the symplectic integrator [11], [12], which conserves energy in the Hamiltonian system. Although the SFDTD scheme has been used to solve the guided-wave [12], electromagnetic radiation, penetration and scattering problems [13], little research has been performed on bioelectromagnetic simulations. Additionally, the traditional FDTD scheme has been used to calculate SARs using the pregnant woman/fetus model [14], [15], but no one has used the SFDTD algorithm to this end. In this article, we apply the high-order SFDTD scheme to a bioelectromagnetic simulation using a simple model of a pregnant woman and her fetus.

In addition, many researchers [14], [16] model SARs, which are limited below the safety guidelines to ensure the safety of the patient, in the human body. A standard [17] has been developed to limit the maximum energy deposition within human subjects undergoing an MRI scan. However, few researchers are concerned with protective measures to reduce the risks posed by RF radiation. For lower SAR distributions at 64 MHz, we choose a 2 cm-deep plasma protective layer to reduce the mother’s/fetus's SARs from a 1.5 T MRI system. By simulating the mother/fetus model with the SFDTD scheme used, we find an optimal angle that not only best protects the patient, but it also significantly reduces the raw material costs.

## Materials and Methods

### Electromagnetic-Field-Solver General Formulations for the SFDTD Scheme

The time-dependent Maxwell’s curl equations in free space are
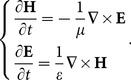
(1)


A helicity Hamiltonian [18], [19] for Maxwell’s equations in a homogeneous, lossless, and sourceless medium can be introduced as
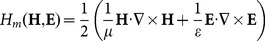
(2)


Where 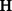 and 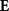 are the magnetic and electric fields. 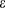 and 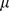 are the permittivity and permeability of the medium. 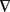 is Curl, which is a vector operator that describes the infinitesimal rotation of a 3-dimensional vector field.

Based on the variational principle, (2) can be rewritten as
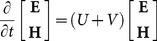
(3)

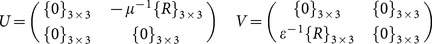
(4)

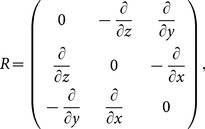
(5)


Where 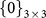 is the 3
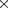
3 null matrix and 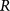 is the 3
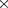
3 matrix representing the three-dimensional curl operator.

Now, Maxwell’s equations with the FDTD scheme applied can be written as an infinite-dimension Hamiltonian system. A stable and accurate solution can be obtained by using the symplectic scheme.

If 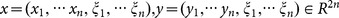, the symplectic inner products of 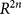 is defined as: 
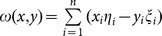
. Meanwhile, if the linear transformation 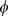 of the symplectic space 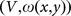 meets 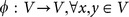, then 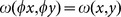. So, 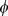 is called a symplectic transformation or a canonical transformation. In other words, the symplectic scheme is just the transformation, in which the symplectic inner products are changeless.

For the temporal direction, the electromagnetic field solution of formula (3) from 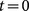 to 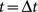 is expressed by using the exponential operator as:
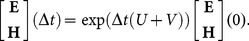
(6)


Where 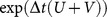 is the time evolution matrix of Maxwell’s equations

Using the product of elementary symplectic mappings, the exact solution (6) can be approximately constructed as [20]:
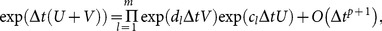
(7)


Where 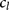 and 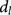 are the constant coefficients of the symplectic integrator. 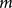 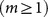 is the stages of the approximation and 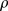 is the order of the approximation. The coefficients can be found by using the Baker-Campbell-Hausdorff (BCH) formula [12], [20], [21], [22].

For the operator 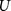 and 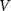 containing the curl operator 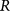, Maxwell’s equations must be discreted in the spatial direction by the higher-order difference for the numerical solutions.

For the spatial direction, the explicit, fourth-order-accurate difference expressions in conjunction with the staggered Yee lattice are used to discretize the first-order spatial derivatives as follows:
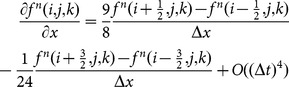
(8)


Where the coefficients 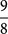 and 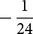 are derivated from the Tayor expanded formula.

So, the relationship between variable 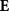 and 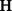 is derived.

As each tissues in the mother/fetus are the dispersive medium, the formula (1) is changed as:
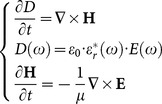
(9)


Where 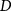 is the electric displacement vector, 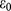 is the permittivity of vacuum, 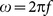, 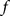 is the frequency of the incident wave and 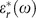 is the complex permittivity of each tissues in the mother/fetus.

Then the normalized electric field 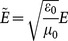 and the electric flux density 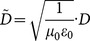 are introduced into the formula (9) as:
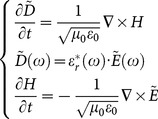
(10)


Where 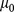 is the permeability of vacuum

For the complex permittivity of each tissues, 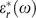 is expressed as
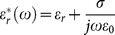
(11)


Where 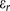 is the averaged relative permittivity of each tissues, 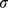 is the conductivity of each tissues.

Substituting the formula (11) into the formula (10):
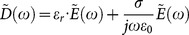
(12)


Then, with the Fourier transform 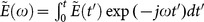, the formula (12) is transformed from the frequency domain to the time domain as
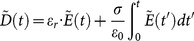
(13)


So, as the integration algorithm is substituted, the formula (13) can be changed with partial summation as:
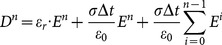
(14)



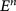
 can be calculated from the formula (14) as
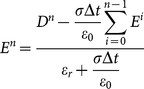
(15)


With 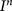 is determined by 
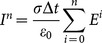
, the formula (15) becomes:
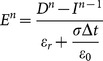
(16a)

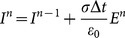
(16b)


So, the relationship between variable 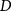 and 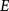 is derived.

From the two-dimensional Maxwell’s equations, the SFDTD scheme, which is fourth-order-accurate in space and fifth-stage-accurate in time, can be obtained by using the discretization approaches above. When 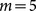, a detailed expression of all the components of the scaled electric field from the 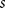-th stage to 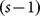-th stage can be written as [12]:
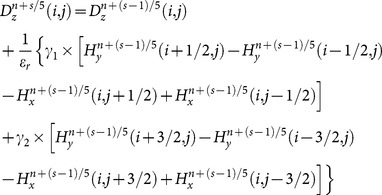
(17)

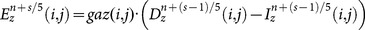
(18)

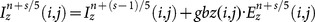
(19)

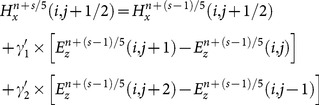
(20)

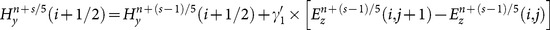


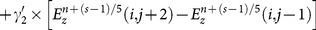
(21)

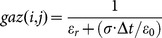
(22a)

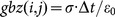
(22b)

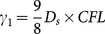
(22c)

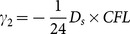
(22d)

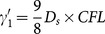
(22e)

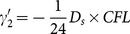
(22f)

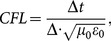
(22g)


Where 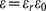 and 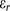 denotes the averaged relative permittivity. 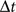 is the time-steps and 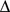 is the length of space-steps. 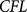 is Courant–Friedrichs–Lewy condition, which is a necessary condition for stability while solving certain partial differential equations numerically by the method of finite differences. Time-steps and space-steps must satisfy the 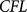 condition. The coefficients 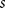 and 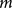 obey the symmetry relations 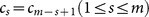, 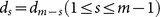 and 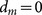.


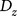
 can be calculated form the formula (17) and 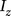 can be calculated form the formula (19). Then 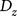 and 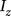 are substituted into the formula (18) to get 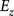. Finally, 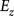 is substituted into the formula (20) and the formula (21) to get 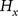 and 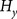 to complete the whole solution of Maxwell’s equations.

### Mother/Fetus Model (Month 9)

A significant amount of research has shown that only a small amount of the body’s tissues (such as the brain) contains magnetic material. In addition, most of the body’s tissues are made of non-magnetic material. The magnetic permeability 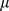 of these tissues is almost 1 and does not need to be considered. For 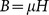, where 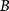 is the magnetic flux density and 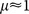, the relationship between 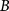 and 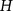 is linear. So only the electric properties of 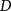 is needed to be considered. Thus, we only address the dielectric properties of the tissues listed in Table 1. To study protective measures for a pregnant woman and her fetus, we developed a simple computational model of a mother and her fetus using the SFDTD scheme because of its high precision and numerical stability. The model used in this article consists of three different types of tissues shown in Table 1: the “uterus”, “placenta” and the “fetus”.

**Table 1:** The dielectric properties of each tissues type, obtained from the materials database, at 64 and 128 MHz.

		64 MHz		128 MHz	
Tissue	Density Values 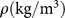	Permittivity 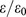	Conductivity 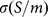	Permittivity 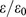	Conductivity 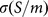
Uterus	1052	92.19	0.91	75.47	0.961
Placenta	1058	86.50	0.95	73.19	1.00
Fetus	987	42.68	0.39	37.60	0.412

**Figure 1:**
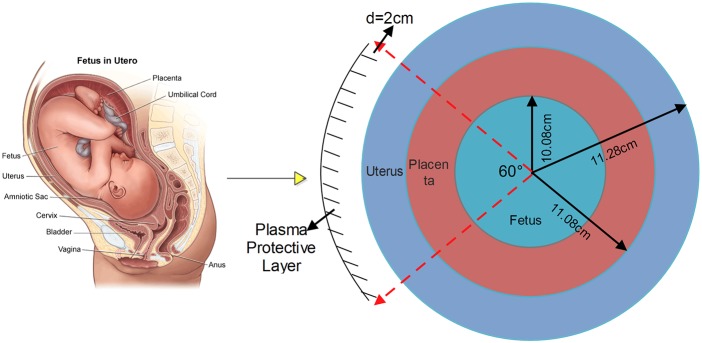
Simple mother/fetus model (month 9) under the plasma protective layer [29].

In Fig. 1, from the numerical model, the radius of the uterus, placenta and fetus are set to 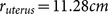, 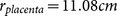, and 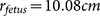 to coincide with their physical dimensions as closely as possible when the fetus is nine months old.

The SFDTD scheme is applied to Maxwell’s equations to calculate the SAR distributions within our model. We establish a computational domain of 450
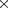
450 cells for this numerical model. Each mother/fetus model consists of a lattice of cubic cells with a length of space-steps 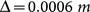, which is sufficient to provide acceptable accuracy for the calculation of the SARs for the fetus. 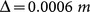 also is the maximum length of the grid, which is used to split the mother/fetus model into a lot of small calculation area. For the 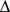 is smaller, the accuracy of the calculation is higher and the 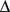 must be less than or equal to 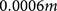. The local SARs are calculated using 800 time-steps iterations.

For harmonically varying fields, the SAR [23], [24], [25], [26] is defined as
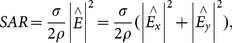
(23)


Where 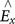 and 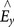 are the peak values of the electric field components and 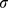 and 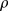 denote the conductivity and the mass density of each tissues, respectively. Because this mother/fetus model is just two-dimensional now, the 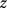 field of 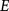 is not to be considered. In the future, the three-dimensional SFDTD scheme will be expanded to the three-dimensional electromagnetic modeling and simulation of the mother/fetus.

A plane wave is incident perpendicular to the protective layer, with frequencies of approximately 64 and 128 MHz to simulate the RF fields of 1.5 T and 3 T MRI systems, respectively.

To ensure the safety of the mother and the fetus, we can add a protective layer where appropriate to reduce the body's SAR distributions [27], [28]. In our realistic model, we choose a plasma material that has a thickness of 2 cm, plasma frequency of 3 GHz and collision frequency of 10 GHz as the protective layer. As shown in Fig. 1, the angle of the plasma protective layer is 60 degree. In addition, the angle may be 0 degree (no protection), 90 degree, 120 degree or 180 degree.

**Figure 2:**
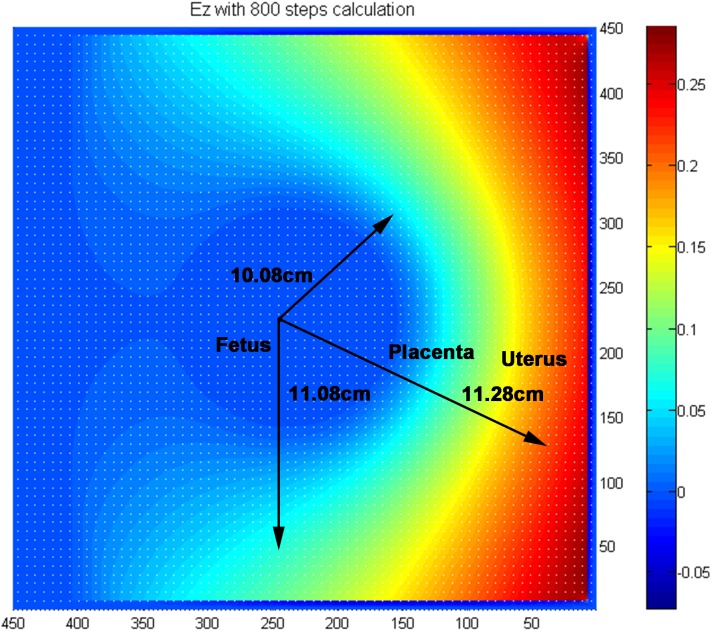
The estimated spatial distributions of the Ez electric field after 800 time-steps iterations using the SFDTD scheme.

The parameter values of the numerical model can be modified according to different requirements. By using 800 time-steps iterations, with an incident 64 MHz plane wave and a protective layer of 60 degree, the results obtained for the 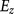 electric field are shown in Fig. 2

## Results and Discussions

### Demonstrating the SFDTD scheme’s benefits

A one-dimensional hard source that uses a Gaussian pulse can be given as.
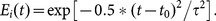
(24)


**Figure 3:**
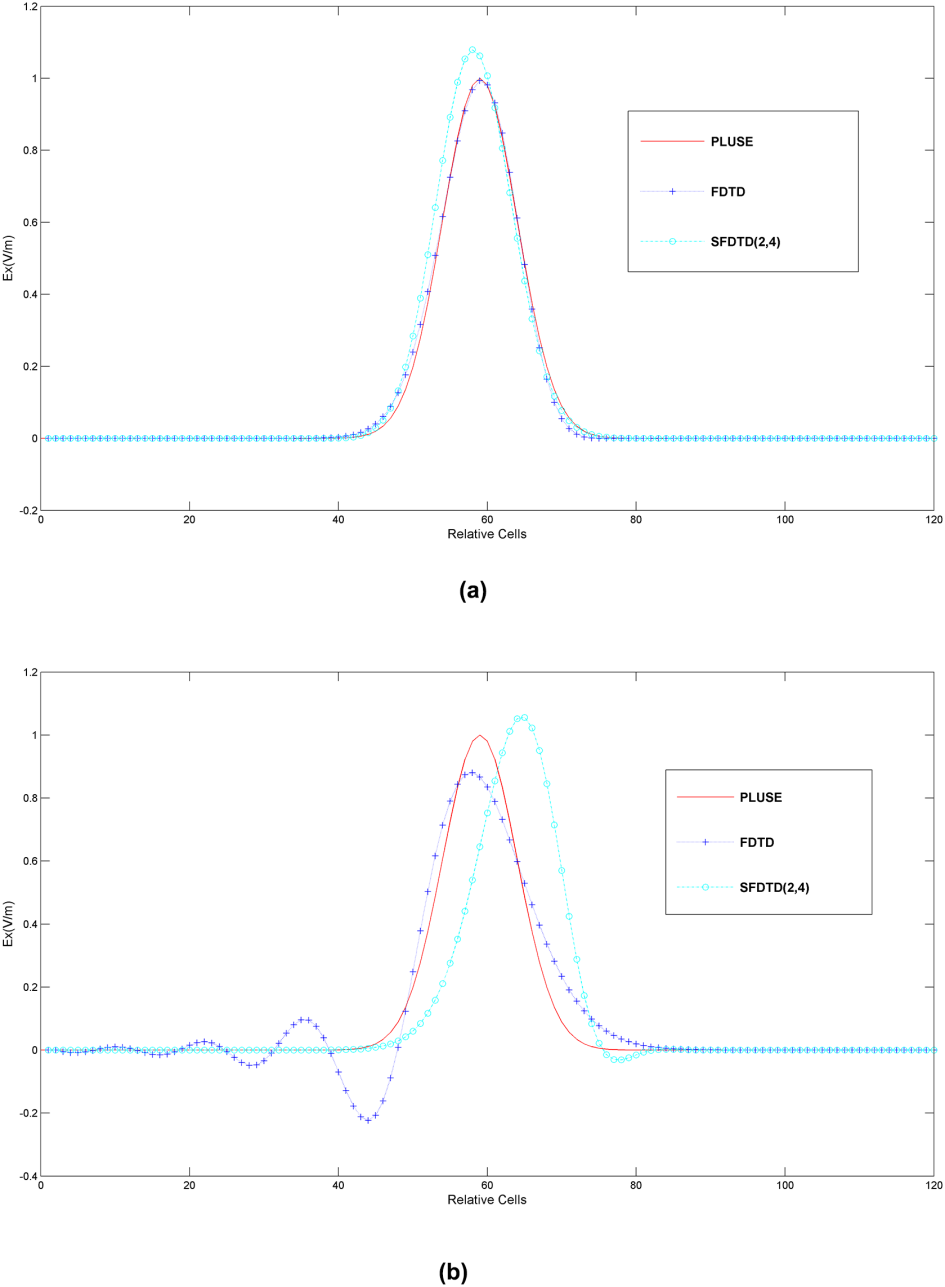
The propagation of the one-dimensional Gaussian pulse for the PEC boundary at (a) 436 time-steps iterations and (b) 3146 time-steps iterations using the SFDTD scheme.

A long-term simulation is performed with 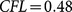 and the length of one space-step 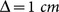, for which is the 10th part of a wavelength. One time-step is 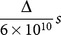. By using the perfect electric conductor (PEC) boundary condition, an one-dimensional resonant cavity is constructed. In Fig. 3, the propagation of the one-dimensional Gaussian pulse is simulated with the analytical solution, the traditional FDTD scheme and the SFDTD scheme respectively at the 436^th^ and 3146^th^ time-steps iteration. Note that Fig. 3 is the one-dimensional solution to prove the SFDTD scheme’s advantage, because only the one-dimensional solution has the analytic solutions to be compared. And the analytic solutions are the curves of the “PLUSE” in Fig. 3. With the number of the time-steps iterations is larger form 436 to 3146, the propagation distance of the pulse is longer.

In Fig. 3, all the algorithms give acceptable results for the pulse calculation with the same waveforms after 436 time-steps iterations. However, at higher iterations, the waveform from the traditional FDTD scheme is greatly distorted after 3146 time-steps iterations with the curve of the traditional FDTD method has been shocked before about 50 relative cells. Where “shocked” means that the curve is not stable and the traditional FDTD method cannot preserve the constant energy of the Hamiltonian system. And relative cells are the relative number of grid. 120 relative cells in Fig. 3 are the size of the computational domain in this simulation.

In addition, the energy of the electromagnetic system computed by the traditional FDTD scheme gradually becomes attenuated. In contrast, under the long-term simulation at the 3146^th^ iteration, the conservation of energy by the SFDTD scheme is verified, as the curve is smooth and in good agreement with the curve of the Gaussian pulse. The peak value of the curve by the SFDTD scheme is always greater than 1 V/m at the 436^th^ iteration or the 3146^th^ iteration. Nevertheless, the peak value of the curve has been less than 1 V/m at the 3146^th^ iteration by the traditional FDTD scheme.

Above all, this demonstrates that the SFDTD scheme can remain stable and accurate by using the symplectic integrator, which preserves the constant energy of the Hamiltonian system. Therefore, the SFDTD scheme can meet the requirements for modeling the mother/fetus model and can calculate the local SARs below with a high precision and in a numerically stable manner.

### The Local SARs using the SFDTD scheme at approximately 64 MHz and 128 MHz with a 60 degree protective layer

**Figure 4:**
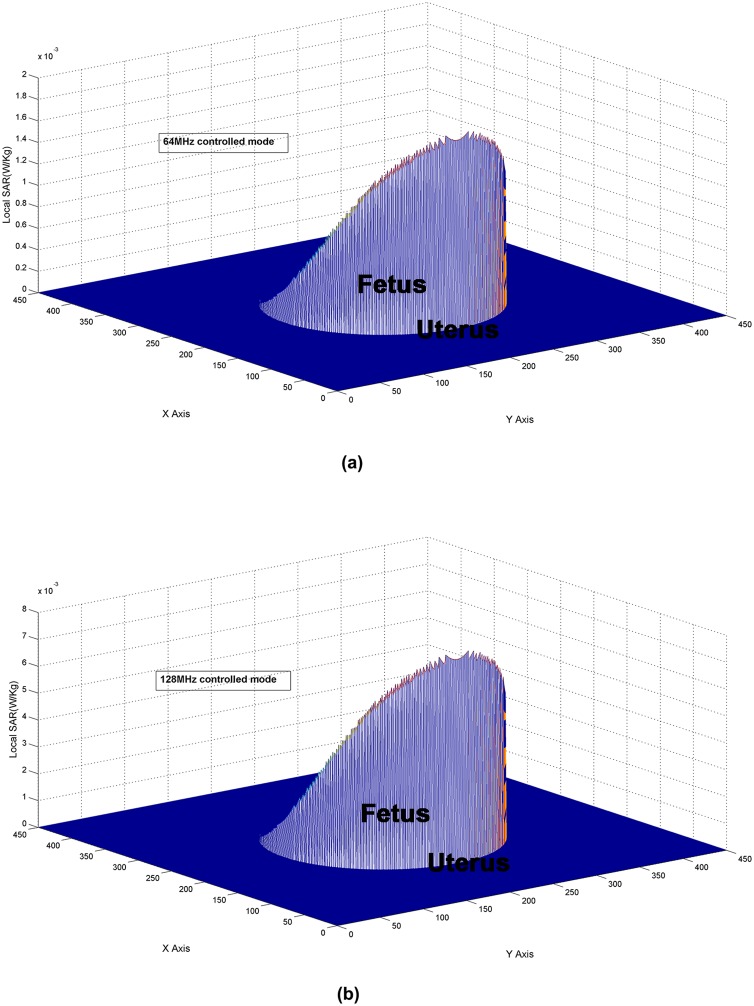
The estimated spatial distributions of the local SARs found by (a) 64 MHz and (b) 128 MHz controlled modes using the SFDTD scheme (the number of the time-steps iterations is 800).

The SAR distributions found on the surface of the mother/fetus model are shown in Fig. 4. Different SAR patterns are observed for 64 and 128 MHz. This is mainly caused by the MRI operating frequency as well as the conductivities used in the mother/fetus models. Our simulation results suggest that higher local SARs are found at 128 MHz rather than at 64 MHz. At 128 MHz, the peak SAR is greater than 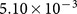
(W/kg), a fourfold increase over the SAR of 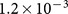
(W/kg) at 64 MHz. In these cases, we choose the 1.5 T MRI system operating at approximately 64 MHz to perform a scan on a pregnant woman because this is safer as a result of the lower SAR distributions.

### The Local SARs using the SFDTD scheme, under the 64 MHz controlled mode, with different plasma protective layer angles

**Figure 5:**
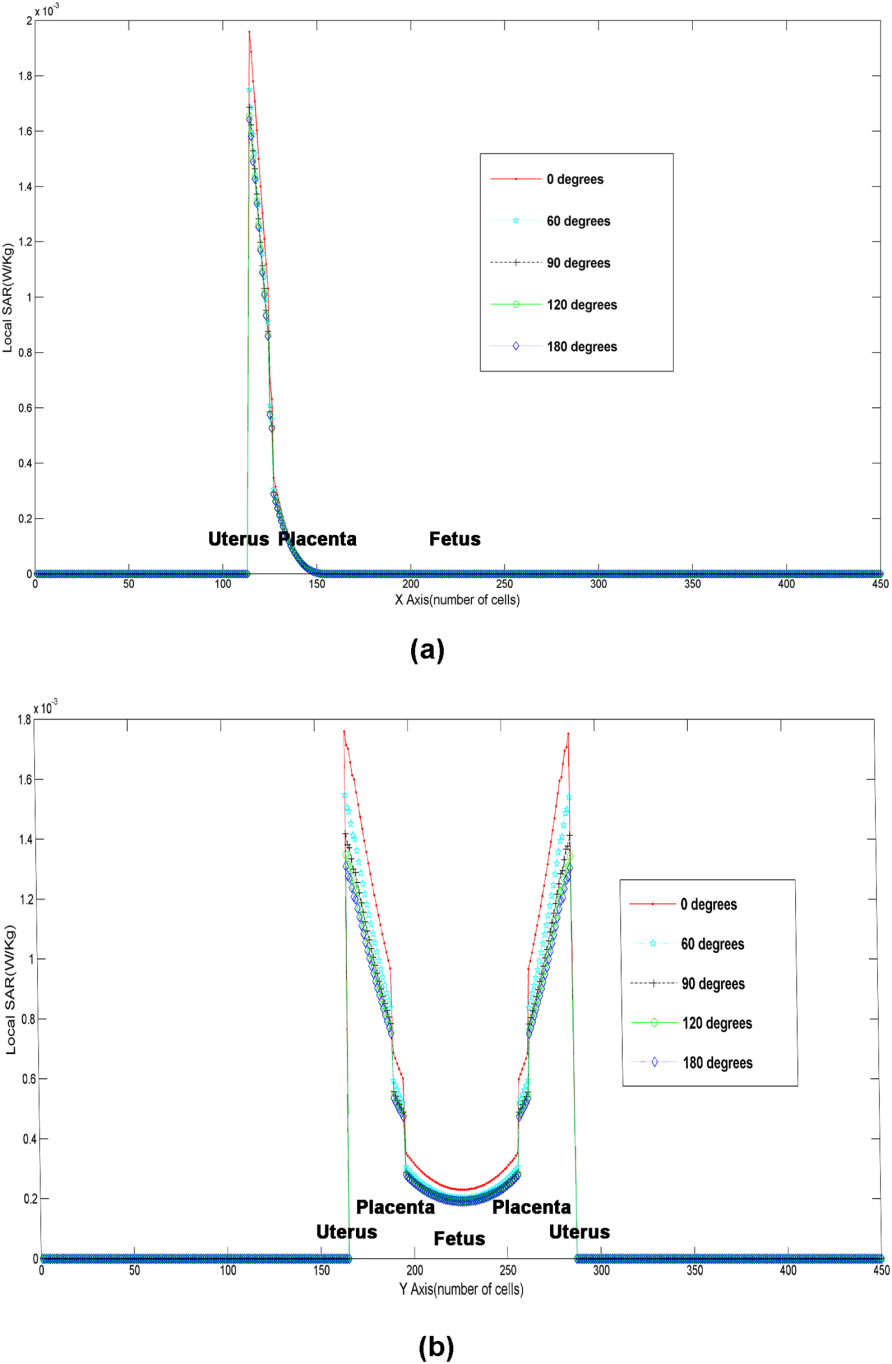
The local SARs in the mother/fetus model on the (a) x-axis and the (b) y-axis using the SFDTD scheme.

In Fig. 5, in comparison to the case of no protective layer, the peak SAR is significantly reduced using a 60 degree plasma protective layer. The maximum value of the SARx is reduced from 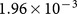
(W/kg) to 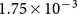
(W/kg), and the maximum value of the SARy is reduced from 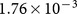
(W/kg) to 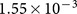
(W/kg) with a 60 degree plasma protection with a reduction of 10 to 12%. It illustrates that the plasma protective layer plays an important role in reducing the electromagnetic radiation received by the mother and fetus.

Additionally, compared to using a 90 degree plasma protection layer, the peak SAR is nearly equal to the 120 degree layer and the 180 degree layer, and it is lower at 60 degree. By increasing the angle of the plasma protective layer from 60 degree to 90 degree, the maximum value of SARx is reduced from 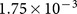
(W/kg) to 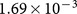
(W/kg), and the maximum value of SARy is reduced from 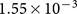
(W/kg) to 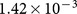
(W/kg) with a reduction of 3.4 to 8.4 per cent. This proves that using a 90 degree plasma protection layer is most effective and economical for the radiation protection of a pregnant woman and her fetus.

## Conclusion

This article derives the SFDTD differential equations and demonstrates the superiority of the SFDTD scheme for researching the mother/fetus model. The SFDTD scheme can make the results more stable and accurate with high precision and meet the requirements for calculating the local SARs of the mother/fetus model. Also, with the SFDTD scheme used, a better frequency of the MRI system and an optimal angle of the plasma protective layer, are found to reduce the values of the local SARs and protect the pregnant women and fetus better.

In addition, not only the SFDTD scheme is better than the traditional FDTD scheme and the high-order FDTD scheme, but also the geometries of the mother/fetus model are relatively simple, therefore the SFDTD scheme is better than the FEM and the FVM for researching the SARs and the optimal angle of the protective layer. Also with the mother/fetus model becoming more and more complex, the symplectic integrator is considered to be added into the FEM or the FVM based on unstructured grids for its advantages in the future research. And the massively parallel computing, which is based on the new algorithm, such as the SFDTD scheme and the FEM above, is considered too. Of course, a more realistic mother/fetus model for electromagnetic simulation will be constructed in the future research. Also in the future, the algorithm, which is used in this article or will be used in the future, will be improved continuously. And some clinical experiments will need to be done to prove our results.

All the forward simulations and calculations are executed on a personal computer with a 2.93 GHz Intel Core2 Duo processor with 2 GBs of RAM using the C/C++ languages. After the calculated data are obtained by the C/C++ codes, the figures above are generated by the Matlab software using these data.
